# Synergistic effects of synbiotic, protein supplementation, and resistance training on inflammation, oxidative stress, and muscle strength in older adults with type 2 diabetes mellitus: a RCT triple-blinded study

**DOI:** 10.1007/s00394-026-04076-7

**Published:** 2026-08-19

**Authors:** Marta Ferreira Bastos, Priscila Larcher Longo, Guilherme Eustáquio Furtado, Joselma Rodrigues dos Santos, Ariana Tito Rodrigues, João Vitor Oliveira Carvalho, Adriana Machado Saldiba de Lima, Guilherme Carlos Brech, André Luis Lacerda Bachi, Marcelo Paes de Barros, Larissa Augusta de Sousa, Rodrigo Assunção de Oliveira, Carolina Rego Chaves Dias, Julia Maria D’Andrea Greve, Angelica Castilho Alonso

**Affiliations:** 1 Postgraduate Program in Aging Sciences, Universidade São Judas Tadeu, Rua Taquari, 546, São Paulo, 03166-000 Brazil; 2 Polytechnic Institute of Coimbra, Applied Research Institute, Coimbra, Portugal; 3 SPRINT - Sport Physical activity and health Research & INnovation cenTer, Polytechnic Institute of Coimbra, Coimbra, Portugal; 4 Laboratory Study of Movement, Instituto de Ortopedia e Traumatologia do Hospital das Clínicas (IOT-HC) da Faculdade de Medicina da Universidade de São Paulo (FMUSP), São Paulo, SP Brazil; 5 Post-Graduation Program in Health Sciences, Santo Amaro University (UNISA), São Paulo, SP Brazil; 6 Ent Research Lab. Department of Otorhinolaryngology - Head and Neck Surgery, Federal University of Sao Paulo, São Paulo, Brazil; 7 Interdisciplinary Program in Health Sciences, Institute of Physical Activity Sciences and Sports (ICAFE), Universidade Cruzeiro do Sul, Rua Galvao Bueno 868, B-Tower, 13th floor, São Paulo, SP 01506-000 Brazil; 8 Post-Graduation Program in Collective Health, Institute of Collective Health, Federal University of Bahia (ISC/UFBA), R. Basílio da Gama, S/N, Campus Universitário Canela, Salvador, Bahia, 40110-040 Brazil

**Keywords:** Aging, Sarcopenia, Inflammation mediators, Exercise, Dietary supplements, Probiotics

## Abstract

**Background:**

Population aging poses major challenges for the management of type 2 diabetes mellitus (T2DM), particularly because inadequate protein intake, physical inactivity, and gut microbial dysbiosis may contribute to systemic oxidative stress and chronic low-grade inflammation (inflammaging). The present study investigated the combined effects of protein supplementation (P), synbiotic plus protein supplementation (SP), and resistance training (RT) on physical performance, body composition, glycemic control, inflammatory and oxidative stress parameters in older adults with T2DM.

**Methods:**

This randomized, triple-blinded clinical trial included men aged ≥ 65 years with T2DM. Fifty-one participants were randomly allocated to one of three intervention groups: Control (*n* = 17), Protein (P; *n* = 17), or Synbiotic + Protein (SP; *n* = 17). Following withdrawals and losses to follow-up, 40 participants completed the intervention and were included in the final analyses (Control, *n* = 13; *P*, *n* = 13; SP, *n* = 14).

**Results:**

All groups showed improvements in physical performance and an enhanced redox balance, as indicated by higher uric acid levels (an endogenous antioxidant) and lower plasma iron concentrations. Although all groups exhibited increased activities of the muscle damage markers lactate dehydrogenase and creatine kinase, the Protein group demonstrated greater improvements in muscle strength, reduced insulin resistance, and lower oxidative damage associated with heme-iron species. Furthermore, the pro-inflammatory cytokines IFN-γ and IL-6 were elevated in the Protein group, which may reflect their physiological role in exercise-induced metabolic adaptation and energy homeostasis rather than a detrimental inflammatory response.

**Conclusions:**

Protein supplementation combined with RT was associated with improvements in muscle strength, physical performance, insulin resistance, and selected biomarkers of oxidative stress. Under the conditions of the present study, the addition of synbiotic supplementation did not provide further benefits beyond those achieved with protein supplementation and resistance training alone.

**Supplementary Information:**

The online version contains supplementary material available at https://doi.org/10.1007/s00394-026-04076-7.

## Introduction

Population aging has been associated with a rising prevalence of type 2 diabetes mellitus (T2DM), with approximately half of individuals with T2DM aged ≥ 65 years. Brazil ranks sixth worldwide, with 15.7 million people affected in 2021 and a projected increase to 23.2 million by 2045, posing substantial societal and public health challenges [1].

T2DM is characterized by insulin resistance and chronic hyperglycemia, leading to traditional complications such as kidney disease, retinopathy, stroke, peripheral neuropathy, coronary artery disease, heart failure, and peripheral vascular disease. More recently, T2DM has also been linked to cancer, infections, liver disease, sleep apnea, affective disorders, and cognitive and functional decline [2]. These complications are mediated by molecular and cellular mechanisms involving increased production of reactive oxygen and nitrogen species, advanced glycation end products, and chronic inflammatory activation [3–6].

Aging and T2DM are independently associated with an increased risk of sarcopenia, highlighting the contribution of diabetes to musculoskeletal dysfunction [7, 8]. Although several mechanisms have been proposed to explain the higher prevalence of sarcopenia in individuals with T2DM, the pathways linking diabetes, muscle loss, and potential mitigating strategies remain incompletely understood [9, 10].

Lifestyle modifications, alongside pharmacological treatment, are essential for improving health outcomes in T2DM, partly through modulation of redox balance and inflammatory status [11, 12]. Current guidelines from the Brazilian Diabetes Society, American Diabetes Association, and European Association for the Study of Diabetes recommend exercise combined with dietary counseling to improve glycemic control [13–15]. Regular physical exercise reduces HbA1c through mechanisms including enhanced mitochondrial density, insulin sensitivity, vascular function, and cardiorespiratory capacity [16, 17].

Dietary interventions combined with exercise improve metabolic control, prevent frailty, and enhance quality of life in older adults with T2DM [18]. Whey protein supplementation has shown benefits for glycemic and cardiometabolic markers in adults with prediabetes; however, its effects in older adults with T2DM remain unclear [19, 20]. Nutritional and lifestyle interventions may also influence the gut microbiota, which plays a central role in host metabolism, immune function, oxidative stress, and anabolic balance [21–27].

Age-related gut dysbiosis contributes to immunosenescence and inflammaging and has been implicated in the pathogenesis of T2DM and sarcopenia [28–31]. Modulation of the gut microbiota using probiotics, prebiotics, synbiotics, or fecal microbiota transplantation has emerged as a potential therapeutic strategy for metabolic and age-related conditions [32–34]. However, no clinical studies have evaluated the combined effects of exercise and synbiotic supplementation in older adults with T2DM. Evidence is currently limited to studies in prediabetes, showing improvements in glycemic control, inflammation, and oxidative stress [35, 36].

Therefore, this study aimed to investigate the combined effects of synbiotic and protein supplementation with resistance training on inflammation, oxidative stress, muscle strength, body composition, and glycemic parameters in older adults with T2DM.

## Methods

### Study design

This randomized, triple-blind clinical trial was conducted between 2022 and 2023 at Universidade São Judas Tadeu (USJT), in collaboration with the Laboratory for the Study of Movement (LSM) at the Institute of Orthopaedics and Traumatology, Hospital das Clínicas, Faculty of Medicine, University of São Paulo (FMUSP), and the Polytechnic Institute of Coimbra (IPC), Portugal. The study adhered to the Consolidated Standards of Reporting Trials (CONSORT) guidelines (Table 1 in Supplementary files) (ClinicalTrials.gov identifier: NCT03792646) and was approved by the Ethics Committees of FMUSP (No. 911.064) and USJT (No. 7.071.72).

### Participants recruitment and settings

Older adults were recruited through advertisements on social media (Facebook, Instagram, and WhatsApp). Eligible participants were community-dwelling men aged 65–79 years with a diagnosis of T2DM, receiving a stable regimen of antidiabetic medication and/or insulin therapy for at least two years, and presenting glycated hemoglobin (HbA1c) concentrations between 6.0 and 9.0%, according to the American Diabetes Association criteria [37].

Eligibility also required preserved renal function, defined as an estimated glomerular filtration rate (eGFR) > 60 mL/min/1.73 m², calculated using the Modification of Diet in Renal Disease (MDRD) equation [38], as well as the absence of musculoskeletal disorders that could limit exercise participation, contraindications to resistance training, or uncontrolled chronic diseases. Participants were excluded if they attended fewer than the minimum required training sessions (i.e., missed more than three sessions) or experienced intervention-related adverse events, including musculoskeletal pain or worsening of pre-existing medical conditions, during the intervention.

### Sample size calculation, randomization, allocation, and blinding

**Fig. 1:**
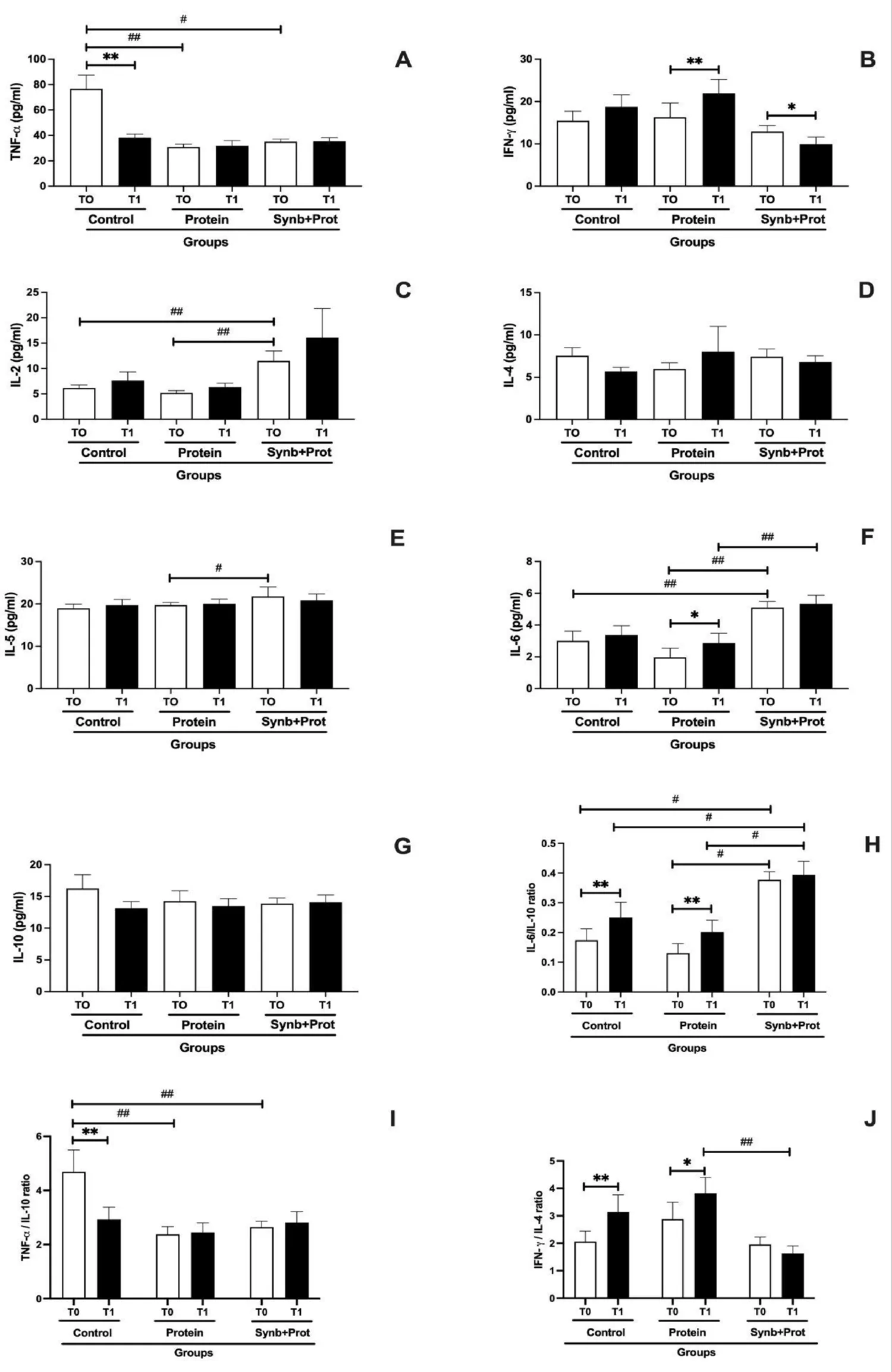
Concentration cytokines before (white columns = T0) and after 12 weeks (black columns = T1) of interventions in the three groups. Data are presented as mean and standard deviation bars. *p < 0.05 and **p ≤ 0.01 represent intragroup differences, while #*p* < 0.05 and ##*p* ≤ 0.01 represent intergroup statistical differences, both detected by generalized linear mixed models (GLMM). **A** Tumor necrosis factor alpha (TNF-α); **B** interferon-gamma (IFN-γ); **C** interleukin (IL) -2; **D** IL-4 **E** IL-5; **F** IL-6; **G** IL-10; **H** IL-6/IL-10 ratio; **I** TNF-α/IL-10 ratio and, **J** IFN-γ/IL-4 ratio

After physician-led screening and eligibility assessment, participants were enrolled according to the predefined sample size calculation [20] and provided written informed consent (Fig. 1, Supplementary File). The a priori sample size calculation indicated that a minimum of 13 participants per group was required [20]. To compensate for anticipated withdrawals and losses to follow-up, 17 participants were recruited per group, resulting in a total of 51 randomized participants. Participants were allocated using a computer-generated randomization sequence to one of three intervention groups: Control (*n* = 17), Protein (*n* = 17), or Synbiotic + Protein (*n* = 17). The randomization process was conducted by an independent investigator who was not involved in participant assessments, intervention delivery, or outcome analyses. After withdrawals and losses to follow-up, 40 participants completed the study and were included in the final analyses (Control, *n* = 13; Protein, *n* = 13; Synbiotic + Protein, *n* = 14), as presented in the CONSORT flow diagram (Fig. 1, Supplementary File).

The study employed a triple-blind design. Outcome assessments were performed by physiotherapists who were blinded to participants’ group allocation and were not involved in the exercise intervention. The exercise intervention was delivered by a physical education professional who remained blinded to the nutritional supplementation assigned to each participant. Nutritional supplements were prepared and dispensed exclusively by the nutrition research team using identical packaging and external appearance for all supplements, ensuring that neither participants nor the exercise intervention provider were aware of whether the assigned supplementation corresponded to the Control, Protein, or Synbiotic + Protein group. Furthermore, statistical analyses were performed using anonymized group codes (Groups A, B, and C), and data analysts remained blinded to treatment allocation until completion of all analyses.

In addition, a bio-sociodemographic questionnaire was used to collect data on age, educational level, marital status, lifestyle behaviors, self-reported health conditions, and medication use.

### Description of interventions

Part of the methods applied in the present study have been previously described elsewhere [20]. All participants received individualized nutritional counseling to standardize daily protein intake according to recommendations of the PROT-AG Study Group for adults aged > 65 years (1.0–1.2 g/kg body weight/day). Adherence to dietary guidance was monitored through regular follow-up visits and dietary assessments conducted by trained nutritionists throughout the intervention period [39].

Maximal muscle strength was assessed using the one-repetition maximum (1RM) test, defined as the maximum load lifted through a complete movement without repetition, as previously described [40]. Exercise intensity was initially set at 70% of 1RM and subsequently adjusted during each session to maintain a perceived exertion of 7–8 on the OMNI Resistance Exercise Scale. Participants completed a 12 week resistance training program consisting of two supervised sessions per week, lasting 45–60 min. Each session included three sets of 8–12 repetitions targeting major muscle groups, with rest intervals of 1–2 min between sets [20].

#### Protein supplementation

Nutritionists administered the supplements immediately after the training session in each group to ensure optimal timing for muscle recovery and synthesis. Participants of protein and synbiotic+protein groups received either 20 g of whey protein (WP), diluted in 150 ml of water, while the control group received 150 ml of water flavored with the same characteristics of the supplements. The dosage of 20 g of whey protein was selected based on previous research demonstrating its efficacy in promoting muscle protein synthesis post-exercise in older adults [41].

#### Symbiotic supplementation

Participants of synbiotic+protein group received commercial capsules of synbiotic containing 10 × 10^10^UFC/ml of *Lactobacillus acidophillus* NCFM, *Lacticaseibacillus paracasei* Lpc-37, *Bifidobactertium lactis* BI-07, *Bifidobacterium lactis* BI-04 and microcrystalline cellulose to be consumed after lunch every day for 12 weeks.

Adherence to the RT program was monitored through supervised session attendance, and participants who missed more than three sessions were excluded. Protein supplementation (or placebo) was administered by the nutrition team immediately after each training session. Adherence to synbiotic supplementation was monitored through supplement diary records maintained by the nutrition team and verified during regular follow-up visits throughout the intervention period.

### Outcomes assessment

All measures described above were performed before (T0) and after 12 weeks (T1) interventions.

#### Body composition

The body composition was determined by Bioelectrical impedance analysis (Biodynamics 450, Seattle, WA, USA) to quantify the body mass, body mass index (BMI), lean mass, Fat mass and body fat percentage (BFP).

#### Muscle strength assessment battery

Handgrip strength test (HGT) was applied using the Jamar^®^ manual dynamometer The testing protocol consisted of three repetitions of three seconds in maximal isometric contractions of the D and ND hands, with a rest period of 60 s. The average was calculated and results were expressed in kilograms of force (kgf) [42].

The Biodex^®^ Multi-Joint System 3 dynamometer (Biodex Medical Systems Inc.) was used to assess knee isokinetic strength. Participants underwent a submaximal warm-up on an ergometric bicycle (five minutes), and were positioned for knee flexion and extension evaluation at 60º/s. Two sets of five repetitions were performed, with a 60-second rest interval, starting with the dominant (D) limb followed by non-dominant (ND). Verbal encouragement was provided to ensure the maximum strength. Evaluation started from 90º of flexion to 20º of extension, with gravity correction according to the manufacturer’s guidelines [43]. Peak torque (PT/BW, in % and normalized for body weight of each participant) and total work (TW, in Joules) were measured in both D and ND limbs of all participants.

Handgrip strength was assessed using a Jamar^®^ handheld dynamometer. Participants performed three maximal isometric contractions lasting three seconds for both dominant (D) and non-dominant (ND) hands, with 60-second rest intervals. Mean values were calculated and expressed in kilograms of force (kgf) [42].

Knee isokinetic strength was evaluated using a Biodex^®^ Multi-Joint System 3 dynamometer (Biodex Medical Systems Inc.). After a five-minute submaximal warm-up on an ergometric bicycle, participants were positioned for concentric knee flexion and extension at an angular velocity of 60°/s. Two sets of five repetitions were performed for each limb, with 60-second rest intervals, starting with the dominant limb. Standardized verbal encouragement was provided to ensure maximal effort. Assessments were conducted through a range of motion from 90° of flexion to 20° of extension, with gravity correction applied according to the manufacturer’s guidelines [43]. Peak torque normalized to body weight (PT/BW, %) and total work (TW, J) were recorded for both limbs.

#### Functional fitness status

The Short Physical Performance Battery (SPPB) was performed to evaluate the static standing balance, usual gait speed, and lower extremity muscle strength. The total score of SPPB ranges from zero (worst) to 12 points (best), providing a comprehensive assessment of functional fitness [44].

#### Advanced glycation end products (AGEs)

The concentration of AGEs was determined non-invasively by measuring skin autofluorescence using the AGE Reader UM^®^ (DiagnOptics, Groningen, The Netherlands), a clinically validated technique that correlates with AGE accumulation measured by skin biopsy [45]. Skin autofluorescence was expressed in arbitrary units (AU) [45].

#### Biochemical analysis

Circulating concentrations of fructosamine, glucose, insulin, glycated hemoglobin (HbA1c), C-peptide, and the homeostatic model assessment for insulin resistance (HOMA-IR) were measured using commercial kits according to the manufacturers’ instructions (Labtest Diagnóstica, Lagoa Santa, MG, Brazil).

Peripheral blood samples (20 mL) were collected in additive-free vacutainer tubes, centrifuged at 3000 rpm for 10 min, and serum was aliquoted and stored at − 80 °C until analysis. Serum concentrations of inflammatory markers (TNF-α, IFN-γ, IL-2, IL-4, IL-5, IL-6, and IL-10) were determined by ELISA using commercial kits (R&D Systems, Minneapolis, MN, USA), following the manufacturers’ protocols.

Redox status was assessed by measuring uric acid, serum iron, heme iron, lactate, lactate dehydrogenase (LDH), and creatine kinase (CK). All spectrophotometric assays were performed in a 96-well microplate reader (SpectraMax M5, Molecular Devices, Sunnyvale, CA, USA) using commercial kits from Bioclin-Quibasa (Belo Horizonte, MG, Brazil: CK NAC #K010, LDH #K014, lactate #K084, UIBC #K211, uric acid #K139) and Labtest Diagnóstica (heme iron #43), according to the manufacturers’.

### Statistical analysis

Data was analyzed by Statistical Package for the Social Sciences (SPSS- 24.0). Normality and homogeneity of variances were assessed using Shapiro-Wilk and Levene’s tests, respectively. Descriptive analysis was conducted using mean and standard deviation for continuous variables. Student’s t-test was employed to compare baseline characteristics of quantitative variables. Categorical variables were presented as absolute and relative values, and their association tested by Chi-square test or Fisher’s exact test. Generalized Linear Mixed Models (GLMM) were applied for data analysis, treating participants as random effects, time as a repeated measure, and group-by-time interactions as fixed effects, and no data imputation was necessary on performing GLMM addressed by missing data. This analysis was conducted to assess time effects, group effects, and the interaction between group and time, with post hoc Bonferroni corrections applied for multiple comparisons. The significance level was established in 5% for all analyses.

## Results

Table 1 summarizes the bio-sociodemographic characteristics and lifestyle behaviors of participants. There was a lower proportion of participants reporting alcohol consumption in the Synbiotic + Protein group (*p* = 0.02). Regarding medications used by participants, there was a higher proportion of self-reported use of sulfonylurea medication (*p* = 0.01) in the Protein group.

**Table 1:** Biosociodemographic characteristics of participants, lifestyle behaviors, self-reported conditions, and medication use across the three groups: control, protein, and synbiotic + protein

Biosociodemographic parameters	Control (*n* = 17)	Protein (*n* = 17)	Synbiotic + protein (*n* = 17)	*p* value
Age (years, M ± SD)	70.1 ± 4.1	70.9 ± 3.6	71.6 ± 4.2	0.50
*Education level* [*n* (%)]				
Higher Education	15 (88.2)	10 (58.8)	12 (70.6)	7.27 (0.29)
High School	0	2 (11.8)	2 (11.8)	
Middle School II	1 (5.9)	5 (29.4)	2 (11.8)	
Middle School I	1 (5.9)	0	1 (5.9)	
*Marital status* [*n* (%)]				
Married	13 (76.5)	12 (70.6)	11 (64.7)	3.29 (0.77)
Single	2 (11.8)	1 (5.9)	1 (5.9)	
Widowed	1 (5.9)	1 (5.9)	3 (17.6)	
Divorced/Separated	1 (5.9)	3 (17.6)	2 (11.8)	
*Income* [*n* (%)] ^#^				
1 to 2 Salaries	4 (23.5)	1 (5.9)	1 (5.9)	5.95 (0.42)
2 to 5 Salaries	4 (23.5)	9 (52.9)	9 (52.9)	
6 to 9 Salaries	4 (23.5)	4 (23.5)	3 (17.6)	
> 10 Salaries	3 (17.6)	3 (17.6)	4 (23.5)	
*Ethnicity* [*n* (%)]				
White	13 (76.5)	15 (88.2)	12 (70.6)	5.85 (0.21)
Mixed/black	0	1 (5.9)	3 (17.6)	
Asian	4 (23.5)	1 (5.9)	2 (11.8)	
*Lifestyle behaviors* [*n* (%)]				
Participation in leisure-time physical activity	7 (41.2)	13 (76.5)	8 (47.1)	4.47 (0.10)
Smoker	1 (5.9)	0	2 (11.8)	3.69 (0.45)
Ex-smoker	6 (35.3)	10 (58.8)	7 (41.2)	
Never smoker	10(58.8)	7(41.2)	8(47.1)	
Alcohol consumption	13 (76.5)	14 (82.4)	7 (41.2)	7.58 (0.02)^*^
*Self-reported conditions* [n (%)]				
Vascular disease	5 (29.4)	3 (17.6)	5 (29.4)	0.82 (0.66)
Retinal disease	2 (11.8)	3 (17.6)	5 (29.4)	1.74 (0.41)
Neuropathy	3 (17.6)	4 (23.5)	3 (17.6)	0.24 (0.88)
Altered foot sensitivity	2 (11.8)	4 (23.5)	2 (11.8)	1.18 (0.55)
Coronary artery disease	6 (35.3)	4 (23.5)	6 (35.3)	0.72 (0.69)
Musculoskeletal pain	10 (58.8)	11 (64.7)	9 (52.9)	0.48 (0.78)
Falls	6 (35.3)	2 (11.8)	2 (11.8)	3.98 (0.13)
Covid-19	3 (17.6)	2 (11.8)	3 (17.6)	0.29 (0.86)
*Medication use* [n (%)]				
Oral hypoglycemic agent				
Biguanides	9 (52.9)	8 (47.1)	9 (52.9)	1.19 (0.55)
Sulfonylurea	5 (29.4)	9 (52.9)	2 (11.8)	13.1 (0.01)^*^
DPP-4 Inhibitor	3 (17.6)	1 (5.9)	3 (17.6)	1.62 (0.44)
Others	7 (41.2)	5 (29.4)	6 (35.3)	4.95 (0.55)
Mixed therapy	4 (23.5)	4 (23.5)	2 (10.5)	1.84 (0.39)
Hypertensive drugs	9 (52.9)	12 (70.6)	7 (41.2)	4.69 (0.09)
Lipid-lowering drugs	6 (35.3)	10 (58.8)	6 (35.3)	2.71 (0.25)
Diuretics	2 (11.8)	6 (35.3)	1 (5.9)	7.04 (0.13)

*Represents statistical significance (*p* < 0.05) detected by Chi-square test or Fisher’s exact test; M ± SD, Mean ± standard deviation; ^#^ Minimum monthly wage in Brazil in 2022 was approximately US$217.00

In terms of body composition, there were no modifications in body composition intragroup analysis (*p* > 0.05). However, differences were detected at T1 moment between Control and Protein groups for fat mass (*p* = 0.015), BMI (*p* = 0.010), and body fat percentage (*p* = 0.017) (Table 2 in Supplementary file).

Muscle strength performance and physical fitness (SPPB) scores at T0 and T1 were presented as the mean values and standard deviations for handgrip strength, peak torque normalized by body weight (PT/BW), and total work (TW) for both dominant (D) and non-dominant (ND) limbs in Table 2.

For handgrip strength in the D and ND limbs, there were no significant time or group effects observed. The Protein group showed significant improvements for PT/BW extensor D (*p* = 0.002) and ND (*p* = 0.003), PT/BW flexor D (*p* = 0.02) and ND (*p* = 0.001), TW in the extensor muscles of the D limb (*p* = 0.008). There were no differences for TW extensor ND, and flexor D and ND (*p* > 0.05) for any group. Regarding physical performance, all groups showed significant improvements in SPPB scores from T0 to T1, indicating enhanced physical function over the intervention period (Table 2).

**Table 2:** Muscle strength performance and short physical performance battery (SPPB) scores at T0 and T1 among the control, protein, and synbiotic + protein groups

Muscle strength parameters (M ± SD)	Control		Protein		Synbiotic + Protein		*p* value
	T0 (*N* = 17)	T1 (*N* = 13)	T0 (*N* = 17)	T1 (*N* = 13)	T0 (*N* = 17)	T1 (*N* = 14)	
Handgrip D (kgf)	39.9 ± 7.7	39.8 ± 7.7	37.5 ± 6.4	37.3 ± 6.5	37.4 ± 8.4	39.7 ± 10.2	0.78
Handgrip ND (kgf)	37.2 ± 8.0	37.4 ± 6.6	35.5 ± 7.1	36.5 ± 7.2	35.2 ± 6.7	37.3 ± 7.3	0.88
PT/BW–Extensor D (%)	155.0 ± 24.7	166.0 ± 22.8	143.0 ± 38.4^b^	175.0 ± 51.6^b^	150.0 ± 35.6	164.0 ± 38.7	^b^0.002*
PT/BW–Extensor ND (%)	159.0 ± 23.6	164.0 ± 13.8	141.0 ± 29.2^b^	175.0 ± 49.1^b^	151.0 ± 32.6	160.0 ± 36.4	^b^0.003*
TW-Extensor D (J)	489.0 ± 83.3	527.0 ± 76.3	485.0 ± 98.2^b^	551.0 ± 75.2 ^b^	481.0 ± 136.0	527.0 ± 123.0	^b^0.008*
TW-Extensor ND (J)	522.0 ± 107.0	549.0 ± 78.5	493.5 ± 91.3	549.5 ± 72.0	484.0 ± 156.0	540.0 ± 131.0	0.31
PT/BW–Flexor D (%)	77.3 ± 18.0	83.9 ± 15.2	74.3 ± 23.7 ^b^	97.5 ± 36.8 ^b^	84.0 ± 28.0	96.7 ± 27.9	^b^0.02*
PT/BW–Flexor ND (%)	73.3 ± 14.1	77.5 ± 15.5	71.1 ± 21.7^b^	94.2 ± 34.0^b^	78.6 ± 24.7	91.5 ± 22.6	^b^0.001*
TW-Flexor D (J)	274.8 ± 82.0	294.7 ± 55.7	262.0 ± 81.4	332.0 ± 57.5	288.0 ± 114.0	332.0 ± 103.0	0.39
TW- Flexor ND (J)	267.0 ± 67.1	267.0 ± 48.8	260.0 ± 73.2	307.0 ± 59.7	284.0 ± 97.9	326.0 ± 92.0	0.48
SPPB	8.8 ± 1.0^a^	10.5 ± 0.9 ^a^	8.6 ± 2.0^b^	10.8 ± 1.3^b^	9.4 ± 1.8^c^	11.0 ± 1.2^c^	^a^0.03 ^b^0.009 ^c^0.004

a, b, and c represent significant within-group differences between T0 and T1 (time effect) for the control, protein, and synbiotic + protein groups, respectively. Statistical analyses were performed using generalized linear mixed models (GLMM). When *p* < 0.05, *p*-values refer to Bonferroni-adjusted post hoc comparisons. M ± SD, mean ± standard deviation; D, dominant; ND, non-dominant; PT/BW, peak torque/body weight; TW, total work; SPPB, short physical performance battery

Table 3 presents the glycemic control parameters at T0 and T1. No significant time, group, or group × time interaction effects were observed for AGE, fructosamine, glucose, insulin, glycated hemoglobin (HbA1c), or C-peptide levels (all *p* > 0.05). In contrast, HOMA-IR showed a significant reduction over time in the Protein group (time effect, *p* = 0.001), and a significant group effect was observed (*p* = 0.03). However, participants in the Protein group presented higher baseline insulin and HOMA-IR values than those in the Control and Synbiotic + Protein groups. Therefore, these findings should be interpreted with caution, as the observed reduction in HOMA-IR may have been influenced, at least in part, by baseline differences between groups and regression to the mean. Notably, no significant group × time interaction was detected for HOMA-IR.

**Table 3:** Glycemic parameters at T0 and T1 among participants allocated to the control, protein, and synbiotic + protein groups

Parameters (M ± SD)	Control		Protein		Synbiotic + protein		*p* -value
	T0 (*N* = 17)	T1 (*N* = 13)	T0 (*N* = 17)	T1 (*N* = 13)	T0 (*N* = 17)	T1 (*N* = 14)	
AGE (AU)	3.0 ± 0.6	2.9 ± 1.1	3.2 ± 1.2	2.4 ± 2.2	3.1 ± 0.6	2.9 ± 1.1	0.54
Fructosamine (µmol/l)	299.0 ± 60.5	306.0 ± 45.1	300.0 ± 51.4	268.0 ± 37.2	277.0 ± 81.9	298.0 ± 67.5	0.27
Glucose (mg/dL)	133.0 ± 39.3	148.0 ± 46.9	139.0 ± 38.2	116.0 ± 27.6	120.0 ± 45.3	123.0 ± 61.0	0.34
Insulin (mU/L)	9.1 ± 4.1	9.6 ± 4.8	18.0 ± 12.1	12.8 ± 7.7	11.1 ± 7.1	14.5 ± 20.4	0.35
HOMA IR	3.1 ± 1.7^#^	3.4 ± 2.1	6.3 ± 4.4^b #^	3.6 ± 2.0^b^	3.3 ± 3.4 ^#^	2.89 ± 2.5	^b^ Time effect = 0.001 ^#^ Group effect = 0.030
HbA1c (%)	7.4 ± 1.0	7.2 ± 0.8	7.1 ± 1.2	6.6 ± 0.8	6.9 ± 1.4	6.8 ± 0.8	0.84
C-Peptide (ng/mL)	2.4 ± 1.0	2.04 ± 1.1	3.7 ± 1.6	2.9 ± 0.9	2.3 ± 1.1	4.0 ± 8.3	0.33

**b** indicates a significant within-group difference between T0 and T1 (time effect) in the Protein group. **#** indicates a significant between-group difference (group effect). Statistical analyses were performed using Generalized Linear Mixed Models (GLMM), followed by Bonferroni-adjusted post hoc comparisons when appropriate. M ± SD = mean ± standard deviation; AGE = advanced glycation end products; AU = arbitrary units; HOMA-IR = homeostatic model assessment of insulin resistance; HbA1c = glycated hemoglobin

Intragroup analysis showed a decreased concentration of TNF-α in the Control group and IFN-γ in Symbiotic+Protein post-intervention (Fig. 1A and B). In addition, an increase of IFN-γ and IL-6 concentration were detected in the Protein group (Fig. 1B and F, respectively). Regarding intergroup analysis, before the intervention (T0), the Control group presented higher TNF-α concentration than other groups (Fig. 1A). Besides, higher IL-2 and IL-6 concentrations were detected in the Symbiotic+Protein group when compared to other groups (Fig. 1C e 1F, respectively), and higher IL-5 concentration in comparison to Protein group in T0 (Fig. 1E). The comparisons after the interventions (T1) showed a higher IL-6 concentration in the Symbiotic+Protein group regarding the Protein group. No differences were observed for IL-4 and IL-10 concentrations after intra e intergroups analysis (Fig. 1D and 1G, respectively).

To investigate if the inflammatory status and Th1/Th2 pattern was influenced by TFR and supplementation, the ratio between IL-6/IL-10, TNF-α/IL-10, IFN-γ/IL-4 was also performed (Figs. 1H–J). Intragroup analysis showed an increased proportion of IL-6/IL-10 (*p* = 0.015 and *p* = 0.035 for Control and Protein group, respectively) and IFN-γ/IL-4 for Control (*p* = 0.008) and Protein group (*p* = 0.033). In addition, there was a decrease of TNF-α/IL-10 ratio for the Control group between T0 and T1 (*p* = 0.001).

Intergroup analysis showed that the Symbiotic+Protein presented a higher ratio of IL-6/IL-10 regarding Control and Protein at T0 (*p* = 0.001 for both) and T1 (*p* = 0.033 for Control and *p* = 0.005 for Protein). Besides, the Control group presented a higher TNF-α/IL-10 ratio at T0 when compared to other groups (*p* = 0.004 for Protein; *p* = 0.008 for Symbiotic+Protein). Regarding IFN-γ/IL-4 ratio, the Protein group exhibited higher value than the Symbiotic+Protein group at T1 (*p* = 0.005).

**Fig. 2:**
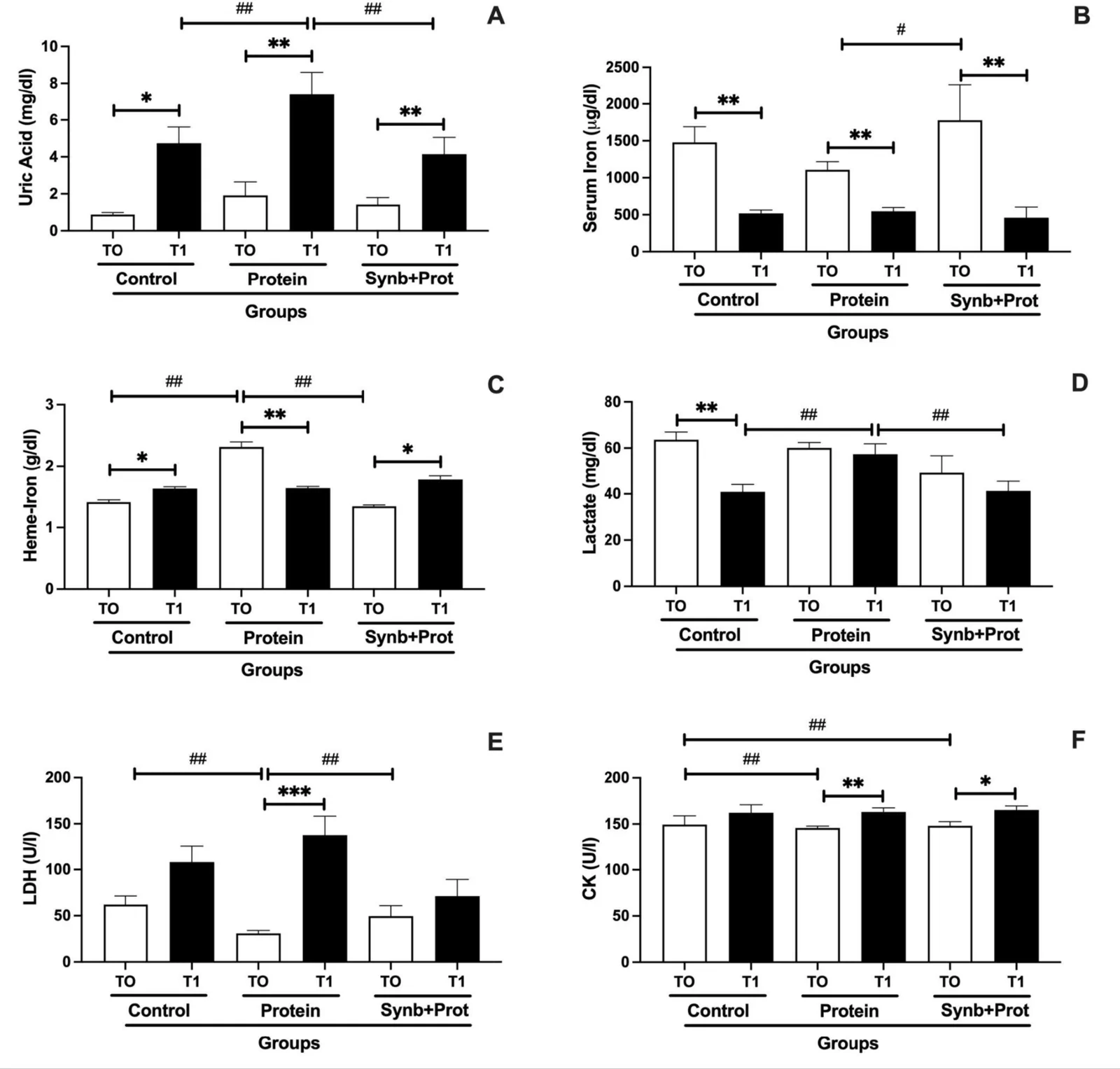
Systemic redox indexes before (white columns = T0) and after 12 weeks (black columns = T1) of interventions in the three groups. Data are presented as mean and standard deviation bars. *p < 0.05 and **p ≤ 0.01 represent intragroup differences, while #*p* < 0.05 and ##*p* ≤ 0.01 represent intergroup statistical differences, both detected by generalized linear mixed models (GLMM). **A** uric acid; **B** serum iron; **C** heme-iron; **D** lactate **E** lactate dehydrogenase (LDH); **F** creatine kinase (CK)

Figures 2A–F present biomarkers of oxidative stress and muscle injury in experimental groups. Regarding muscle injury indexes, slight T0 differences were observed among groups, mostly < 10%, except for LDH activities between Control and Protein, which presented a reduction of 45%. In general, the Control group showed higher levels of muscle injuries than other groups at T0, based on both LDH and CK activities (Fig. 2E and 2F, respectively). Despite the slight T0 differences, data here show that the strength exercise intervention increased muscle injury, especially when evaluated by plasmatic LDH activities (e.g. T1 3.2-fold higher than T0 in Protein group; Fig. 2E), and plasmatic CK activities [T0 vs. T1, in both Protein (+ 11%) and Synbiotic+Protein groups (+ 9%); Fig. 2F]. Figure 2D shows unaltered levels of plasmatic Lactate in both Protein and Synbiotic+Protein groups after RT, whereas strength exercises alone (thus, Control group) promoted a significant reduction of lactate (37%; Fig. 2D). All experimental groups showed higher levels of Uric Acid at T1 (average of 2.7-fold higher in all groups; Fig. 2A), which approximately matches the observed decrease in Serum Iron concentrations (overall reduction of 65%, approximately, considering T0 vs. T1, in Fig. 2B). On the other hand, Heme-Iron concentrations showed more distinguished results: Protein group showed a reduction of 35% at T1, whereas Control and Synbiotic+Protein showed increases of 10% and 38%, respectively (Fig. 2C).

## Discussion

The initial hypothesis of this study was that synbiotic plus protein supplementation, combined with resistance training, would enhance the metabolic and functional benefits of resistance training by improving protein utilization through the modulation of gut dysbiosis, a common feature in older adults with T2DM. Specifically, we hypothesized that this combined intervention would improve glycemic control, attenuate inflammation and oxidative stress, and promote greater gains in muscle strength and physical function compared with protein supplementation or resistance training alone.

The study population was predominantly composed of community-dwelling older men living in an urban region of São Paulo, with relatively high educational attainment and socioeconomic status. These characteristics should be considered when interpreting the findings, as socioeconomic conditions are known to influence lifestyle, healthcare access, and long-term metabolic health, potentially limiting the generalizability of the results to the broader Brazilian older population [46, 47].

At baseline, the intervention groups were generally comparable regarding demographic and clinical characteristics. Although participants in the Synbiotic + Protein group reported lower alcohol consumption, the Protein group presented a higher prevalence of sulfonylurea use, which may partially explain their higher body mass index, fat mass, and body fat percentage at baseline [48].

Overall, the present findings demonstrate that RT promoted beneficial adaptations regardless of supplementation strategy, including improvements in physical performance, reduced serum iron concentrations, and increased uric acid levels, suggesting an enhanced antioxidant status. The Protein group exhibited the most consistent metabolic and functional improvements, including greater muscle strength, lower insulin resistance (HOMA-IR), and reduced heme-iron concentrations, despite increases in CK, LDH, IL-6, IFN-γ, and lactate, which likely reflect exercise-induced metabolic and muscular adaptations rather than adverse responses. In contrast, the addition of synbiotic supplementation did not confer additional benefits beyond those observed with protein supplementation and resistance training. Although the Control group showed lower lactate concentrations and a reduction in TNF-α, the interpretation of these findings should be made cautiously because baseline TNF-α concentrations were approximately 2.5-fold higher than those observed in the other intervention groups.

### Muscle strength, fitness performance and body composition

As hypothesized, resistance training improved physical performance across all intervention groups, whereas greater gains in muscle strength were observed primarily in the Protein group. These findings are consistent with previous studies reporting superior improvements in muscle strength following protein supplementation combined with RT in older adults [49, 50]. Although between-group differences did not reach statistical significance, the direction of the observed effects suggests that protein supplementation may enhance the adaptive response to RT. Future studies should investigate whether higher protein doses or longer intervention periods produce clinically meaningful benefits in this population [51].

No significant changes in body composition or muscle strength were observed over time in the Control and Synbiotic + Protein groups. Nevertheless, all groups demonstrated significant improvements in physical performance, as assessed by the Short Physical Performance Battery (SPPB), reinforcing the well-established role of resistance training in preserving functional capacity among older adults with T2DM. Given that lower SPPB scores are strongly associated with mobility limitations, disability, falls, and loss of independence, these findings suggest that RT remains an effective strategy for maintaining functional health in this population, regardless of the nutritional supplementation provided [52, 53].

### Glycemic control parameters

Improving glycemic control remains one of the primary therapeutic goals in older adults with T2DM. In addition to resistance training, several nutritional interventions—including vitamin D, whey protein, antioxidants, creatine, L-arginine, leucine-rich amino acids, broccoli sprout extract, probiotics, and synbiotics—have been investigated as complementary strategies to optimize glucose metabolism [49, 54, 55]. A recent meta-analysis reported that probiotics and synbiotics, particularly multi-strain formulations, may contribute to modest improvements in glycemic control, including reductions in HbA1c [55]. However, most available evidence has been derived from younger or middle-aged adults, limiting its applicability to older individuals with long-standing T2DM.

In the present study, neither protein nor synbiotic supplementation produced significant changes in fasting glucose, fructosamine, insulin, HbA1c, or C-peptide concentrations. Although participants allocated to the Protein group presented higher HOMA-IR values at baseline, insulin resistance decreased following the intervention to levels comparable with those observed in the Control and Synbiotic + Protein groups. These findings suggest a potential improvement in insulin sensitivity among participants receiving protein supplementation, although this effect should be interpreted cautiously because of baseline between-group differences.

Our findings are partially consistent with those reported by Miller et al. [49], who also observed no significant between-group differences in glycemic control or insulin resistance after 24 weeks of RT combined with protein and vitamin D supplementation in obese older adults with T2DM. Collectively, these findings indicate that RT alone may contribute to maintaining glycemic stability in this population, whereas additional nutritional supplementation appears to exert only modest effects under the intervention conditions evaluated in the present study.

### Inflammatory and redox biomarkers

One of the most relevant findings of the present study was the improvement in redox-related biomarkers following resistance training, particularly among participants receiving protein supplementation. All groups exhibited increased uric acid concentrations and reduced serum iron levels after the intervention, suggesting an enhanced systemic antioxidant response. In contrast, heme-iron concentrations decreased only in the Protein group, whereas they increased in the Control and Synbiotic + Protein groups. Because free iron and heme-derived iron are potent pro-oxidant molecules capable of promoting reactive oxygen species generation, these findings suggest that protein supplementation may have contributed to a more favorable redox environment in older adults with T2DM [56].

Regarding inflammatory biomarkers, the reduction in TNF-α observed in the Control group is consistent with the well-established anti-inflammatory effects of regular resistance training [57]. Participants receiving protein supplementation exhibited increased circulating IL-6 and IFN-γ concentrations together with higher CK and LDH activities and greater improvements in muscle strength. Rather than indicating a detrimental inflammatory response, these findings are more likely to reflect physiological adaptations to resistance exercise. In particular, IL-6 is now recognized as a myokine involved in regulating substrate availability, muscle remodeling, and post-exercise metabolic adaptation, while transient increases in CK and LDH are expected markers of exercise-induced muscle remodeling [58–60].

Previous studies evaluating protein supplementation in older adults with T2DM have generally reported modest or no effects on inflammatory markers [49, 61, 62]. Conversely, some evidence suggests that higher protein intake may influence circulating IL-6 concentrations depending on protein source, dose, and metabolic status [63, 64]. Therefore, the present findings support the hypothesis that protein supplementation may potentiate the physiological adaptations induced by RT rather than promoting chronic systemic inflammation.

In contrast, the addition of synbiotic supplementation did not provide further improvements in inflammatory or redox biomarkers. This finding should be interpreted cautiously, as the lack of effect may be related to the specific bacterial strains used (*Lacticaseibacillus acidophilus*,* Lacticaseibacillus paracasei*, and *Bifidobacterium lactis*), the administered dose (10 × 10¹⁰ CFU/day), or the 12-week intervention period. Furthermore, no microbiome analyses were performed, precluding confirmation that the synbiotic effectively modified gut microbial composition or function.

### Strengths and limitations

The present randomized controlled trial has several strengths. To our knowledge, this is among the first studies to investigate the combined effects of resistance training with protein and synbiotic supplementation on muscle strength, glycemic control, inflammatory biomarkers, and redox status in community-dwelling older adults with established T2DM. The randomized design, supervised exercise protocol, comprehensive biochemical assessments, and repeated measurements over time strengthen the internal validity of the findings. Furthermore, the use of generalized linear mixed models enabled the analysis of repeated measures while accounting for within-subject correlations and missing observations without requiring data imputation.

Nevertheless, several limitations should be acknowledged. First, the 12-week intervention period may have been insufficient to detect longer-term adaptations, particularly those related to synbiotic supplementation. Second, participants presented heterogeneous clinical characteristics, including differences in T2DM duration, medication use, comorbidities, and baseline inflammatory and redox status, which may have influenced individual responses to the interventions. Third, although the final sample size satisfied the a priori calculation for the primary outcome, participant attrition may have reduced statistical power for secondary outcomes. In addition, no sensitivity analysis was performed to evaluate the potential impact of participant dropout. Finally, although modulation of the gut microbiota was the primary rationale for synbiotic supplementation, no microbiome-related analyses were performed, preventing confirmation that the intervention effectively altered gut microbial composition or function.

### Future research

Future randomized controlled trials should investigate longer intervention periods, larger sample sizes, and different synbiotic formulations, including alternative bacterial strains, dosages, and delivery strategies. The incorporation of microbiome-related outcomes, such as gut microbial composition, short-chain fatty acid production, and metabolomic profiling, would improve understanding of the mechanisms underlying synbiotic supplementation in older adults with T2DM. In addition, future studies should consider stratifying participants according to disease duration, medication use, and baseline metabolic or inflammatory status to identify subgroups that may derive greater benefit from nutritional interventions combined with RT.

### Practical implications

The present findings reinforce resistance training as an effective non-pharmacological strategy for improving physical performance in older adults with T2DM. Protein supplementation may provide additional benefits for muscle strength and selected metabolic adaptations when combined with resistance training. Conversely, under the specific conditions evaluated in this study, synbiotic supplementation did not confer additional clinical benefits. These findings suggest that resistance training should remain the cornerstone of exercise-based management for older adults with T2DM, whereas protein supplementation may be considered as an adjunctive nutritional strategy. The routine use of synbiotic supplementation for this population cannot be recommended based on the present findings and requires further confirmation through larger and longer-term clinical trials.

## Conclusion

Resistance training improved physical performance in older adults with T2DM regardless of the nutritional intervention. Protein supplementation provided additional benefits by enhancing muscle strength and promoting favorable changes in selected redox biomarkers and insulin resistance. In contrast, synbiotic supplementation did not confer additional benefits beyond those achieved with resistance training and protein supplementation under the specific formulation, dose, and 12-week intervention period evaluated in this randomized controlled trial.

## Supplementary Information

Below is the link to the electronic supplementary material.

Supplementary Material 1.

## Data Availability

All data supporting the results of this study are included in the article and its Supplementary Information and are available for consultation as needed.
